# The Effect of Thermophoresis on Unsteady Oldroyd-B Nanofluid Flow over Stretching Surface

**DOI:** 10.1371/journal.pone.0135914

**Published:** 2015-08-27

**Authors:** Faiz G. Awad, Sami M. S. Ahamed, Precious Sibanda, Melusi Khumalo

**Affiliations:** 1 Department of Mathematics, University of Johannesburg, P.O. Box 17011, Doornfontein 2028, South Africa; 2 University of KwaZulu-Natal, School of Mathematics, Statistics and Computer Science, Private Bag X01, Scottsville, Pietermaritzburg 3209, South Africa; Massachusetts Institute Of Technology, UNITED STATES

## Abstract

There are currently only a few theoretical studies on convective heat transfer in polymer nanocomposites. In this paper, the unsteady incompressible flow of a polymer nanocomposite represented by an Oldroyd-B nanofluid along a stretching sheet is investigated. Recent studies have assumed that the nanoparticle fraction can be actively controlled on the boundary, similar to the temperature. However, in practice, such control presents significant challenges and in this study the nanoparticle flux at the boundary surface is assumed to be zero. We have used a relatively novel numerical scheme; the spectral relaxation method to solve the momentum, heat and mass transport equations. The accuracy of the solutions has been determined by benchmarking the results against the quasilinearisation method. We have conducted a parametric study to determine the influence of the fluid parameters on the heat and mass transfer coefficients.

## Introduction

A wide variety of fluids, such as polymer solutions, plastics, pulps, emulsions, blood plasma, chocolate, tomato sauce, mustard, mayonnaise, toothpaste, asphalt, some greases and sewage, petroleum products, oils, etc., are non-Newtonian in character. Such fluids have a non-linear shear stress-strain rate relationship. The equations that model the flow of these fluids are generally of a higher order than the Navier-Stokes equations. During the last several decades, the study of flow of non-Newtonian fluids, has received considerable amount of research interest, due to the relevance of non-Newtonian flows to a large number of engineering and manufactural applications such as in the processing of synthetic fibers, food, polymers melts and pharmaceutical products.

The unsteady boundary layer flow of non-Newtonian fluids due to stretching surface is an important field of research in fluid mechanics. In many practical problems, the flow could be unsteady due to a time dependent free stream velocity. There are several transport processes with surface mass transfer where the buoyancy force arises from thermal diffusion caused by the temperature gradient. It is interesting as well as useful to investigate the combined effects of thermal diffusion and surface mass transfer on a stretching surface where the free stream velocity varies arbitrarily with time. The stretched boundary layer flow with heat transfer has numerous applications in engineering and industrial processes such as when polymer sheets are extruded continuously from a dye, in the annealing and thinning of copper wires, paper production and glass blowing, aerodynamic extrusion of plastic sheets and the cooling of infinite metallic plates in cooling baths. Mixed convection or buoyancy driven flows over a surface occur naturally in geothermal and petroleum recovery processes, solid matrix heat exchanges, the cooling of nuclear reactors and other practical problems. Historically, the study of a boundary layer flow along a stretching surface has its origins in the work of Sakiadis [1]. Tsou et al. [2] presented a combined experimental and analytical study of the stretching flow, which, in essence demonstrated that such a flow is physically possible. Crane [3] further extended the Sakiadis study to a linearly stretching plate in a quiescent fluid and presented an exact analytic solution. Subsequent work has looked at various aspects of the stretching sheet problem, but most have been concerned with how the flow is affected by or responds to changes in various fluid and surface parameters, see for instance, [4–9].

Conventional fluids such as water, ethylene glycol and oil have low heat transfer characteristics owing to their low thermal conductivity. A recent technique to enhance the thermal conductivity of these base fluids is to suspend nano-sized metallic particles such as aluminum, titanium, gold, copper, iron or their oxides in the conventional base fluids resulting in what has come to be name as a “nanofluid,” Choi [10]. Nanofluids have considerably improved thermo-physical properties such as thermal conductivity, thermal diffusivity, viscosity and convective heat transfer coefficient compared to base fluids. In the last few years the flow of nanofluid through different geometries, and under various physical assumptions have been studied by several authors such as [11–14].

The Oldroyd-B constitutive model describes a subclass of non-Newtonian fluids that adequately describe the behaviour of some viscoelastic fluids such as dilute polymer solutions. The Oldroyd-B fluid can describe stress-relaxation, creep and the normal stress differences but it cannot describe either shear thinning or shear thickening, a phenomenon that is exhibited by many polymer materials. Nonetheless, this model is perhaps one of the most successful models for describing the response of some polymeric liquids [15]. Some investigations of Oldroyd-B fluids have been done, by, among others, Hayat et al. [16] who presented a study of the three-dimensional flow of an Oldroyd-B fluid due to a stretching surface with convective boundary conditions. Siddiqui et al. [17] investigated the unsteady flow of an incompressible Oldroyd-B fluid between two infinite parallel plates subject to slip between the plates and the fluid. Jamil et al. [18] further studied the unsteady flow of an Oldroyd-B fluid and solved the model equations using finite Hankel transforms. Sohail et al. [19] investigated the two-dimensional steady incompressible Oldroyd-B nanofluid flow past a stretching sheet. The thermophoresis and radiation effects on Heat and mass transfer characteristics in three-dimensional flow of an Oldroyd-B fluid due to a bi-directional stretching surface were investigated by Shehzad et al. [20]. Related studies include, among others, [21–25]. Excellent survey papers on Oldroyd-B fluids can be found in studies by Rajagopal and his colleagues [26–28].

There are currently only a few theoretical studies on the convective boundary layer flow and heat transfer in an Oldroyd-B nanofluid over a stretching surface. Including nanoparticles in dilute polymer solutions imparts improved thermophysical properties to the polymer materials. This could, for example, include improved electrical, mechanical and optical properties, Chao [29]. Recent exception is the study by Khan et al. [30]. These studies all assumed that the nanoparticle volume fraction at the boundary is actively controlled. To the best of the authors’ knowledge, there are as yet no studies of convective heat transport in polymer nanocomposites such as an Oldroyd-B nanofluid flow in which the nanofluid particle fraction on the boundary is not actively managed. The objective of this study is therefore to investigate thermophoresis effects in unsteady Oldroyd-B nanofluid flow along a stretching surface. The problem is formulated under the assumption that the nanoparticle volume fraction at the boundary is not actively controlled, see Nield and Kuznetsov [31–34]. The highly non-linear momentum, heat and mass transfer equations are solved numerically using the spectral relaxation and quasi-linearization methods, see Motsa et al. [35, 36].

## 1 Mathematical Formulation

**Fig 1:**
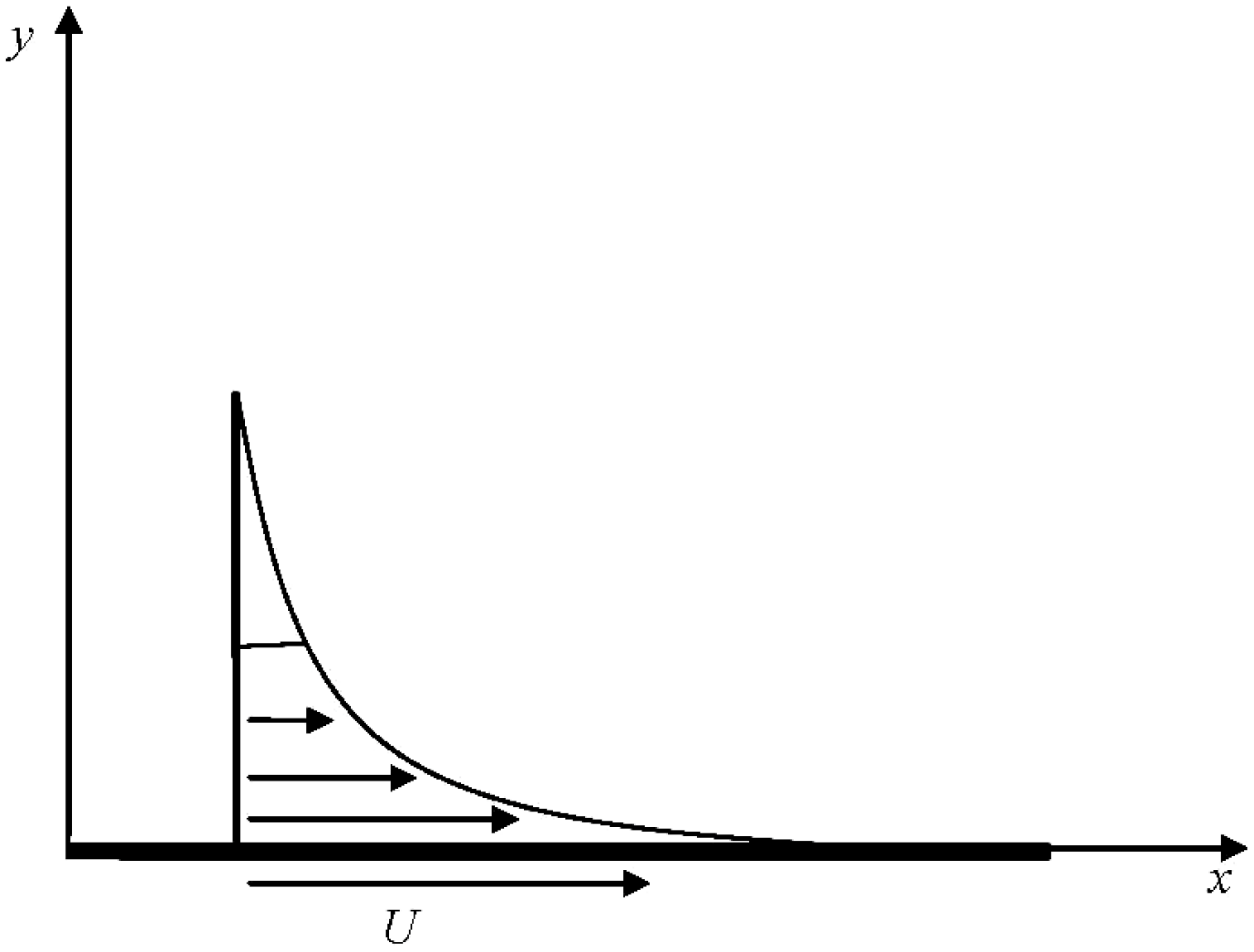
Geometry and the coordinate system.

Consider the unsteady two-dimensional Oldroyd-B nanofluid flow over a stretching surface. The sheet stretches along the plane *y* = 0 with the flow confined in the region *y* > 0. The nanoparticle flux at the boundary surface is assumed to be zero. The surface is stretched with a velocity U = *bx*/(1−*at*) where *a* and *b* are positive constants. Both the nanofluid and the surface are kept at a constant temperature *T*_*w*_ where *T*_*w*_ > *T*_∞_ is for a heated surface and *T*_*w*_ < *T*_∞_ corresponds to a cooled surface. The geometry of the problem is shown in Fig 1. Applying the Oberbeck-Boussinesq and the boundary layer approximations to the basic equations of an incompressible non-Newtonian fluid, we obtain

$$\begin{array}{c} \nabla \cdot V=0, \end{array}$$ (1)

$$\begin{array}{c} \rho \frac{dV}{dt}=\nabla \cdot \tau , \end{array}$$ (2)

$$\begin{array}{ccc} {\left(\rho c\right)}_{f}(\frac{\partial T}{\partial t}+V\cdot \nabla T) & = & {\left(\rho c\right)}_{p}[D_{B}\nabla C\cdot \nabla T+(\frac{D_{T}}{T_{\infty }})\nabla T\cdot \nabla T]\\ & & +k\nabla^{2}T, \end{array}$$ (3)

$$\begin{array}{c} \frac{\partial C}{\partial t}+V\cdot \nabla C=D_{B}\nabla^{2}C+(\frac{D_{T}}{T_{\infty }})\nabla^{2}T. \end{array}$$ (4)

The Cauchy stress tensor *τ* and extra stress tensor **S** are defined as

$$\begin{array}{c} \tau =-pI+S\;\text{and}\;S+{\hat{\delta }}_{1}\left(t\right)\frac{DS}{Dt}=\mu (A+{\hat{\delta }}_{2}\left(t\right)\frac{DA}{Dt}), \end{array}$$ (5)

where

$$\begin{array}{c} \frac{D}{Dt}=\frac{\partial (\cdot )}{\partial t}+\left(V\cdot \nabla \right)\left(\cdot \right)-\left(\nabla V\right)\left(\cdot \right)-\left(\cdot \right){\left(\nabla V\right)}^{*}, \end{array}$$

is the covariant differentiation, the * denotes the matrix transpose, ${\hat{\delta }}_{1}(t)$ and ${\hat{\delta }}_{2}(t)$ are the relaxation and retardation times respectively. The velocity field is

$$\begin{array}{c} V=(u(x,y,t),v(x,y,t)), \end{array}$$

where *u* and *v* are the velocity components along the *x* and *y* directions respectively. The first Rivlin-Ericksen tensor **A** is defined as

$$\begin{array}{c} A=\nabla V+{\left(\nabla V\right)}^{*}. \end{array}$$

The governing equations take the form (see Nadeem et al. [19])

$$\begin{array}{c} \frac{\partial u}{\partial x}+\frac{\partial v}{\partial y}\;=\;0, \end{array}$$ (6)

$$\begin{array}{ccc} & & \frac{\partial u}{\partial t}+u\frac{\partial u}{\partial x}+v\frac{\partial u}{\partial y}+{\hat{\delta }}_{1}\left(t\right)[\frac{\partial^{2}u}{\partial t^{2}}+\frac{\partial }{\partial t}(u\frac{\partial u}{\partial x}+v\frac{\partial u}{\partial y})+u\frac{\partial^{2}u}{\partial t\partial x}+v\frac{\partial^{2}u}{\partial t\partial y}\\ & & \;\;\;-\frac{\partial u}{\partial t}\frac{\partial u}{\partial x}-\frac{\partial v}{\partial t}\frac{\partial u}{\partial y}+u^{2}\frac{\partial^{2}u}{\partial x^{2}}+v^{2}\frac{\partial^{2}u}{\partial y^{2}}+2uv\frac{\partial^{2}u}{\partial x\partial y}]=\nu \frac{\partial^{2}u}{\partial y^{2}}\\ & & \;\;\;+\nu {\hat{\delta }}_{2}\left(t\right)[\frac{\partial^{3}u}{\partial t\partial y^{2}}+u\frac{\partial^{3}u}{\partial x\partial y^{2}}+v\frac{\partial^{3}u}{\partial y^{3}}-\frac{\partial u}{\partial x}\frac{\partial^{2}u}{\partial y^{2}}-\frac{\partial u}{\partial y}\frac{\partial^{2}v}{\partial y^{2}}],\\ & & \frac{\partial T}{\partial t}+u\frac{\partial T}{\partial x}+v\frac{\partial T}{\partial y}=\alpha_{m}\frac{\partial^{2}T}{\partial y^{2}}+\tau [D_{B}\frac{\partial C}{\partial y}\frac{\partial T}{\partial y}+\frac{D_{T}}{T_{\infty }}{\left(\frac{\partial T}{\partial y}\right)}^{2}], \end{array}$$ (7)

$$\begin{array}{c} \frac{\partial C}{\partial t}+u\frac{\partial C}{\partial x}+v\frac{\partial C}{\partial y}=D_{B}\frac{\partial^{2}C}{\partial y^{2}}+\frac{D_{T}}{T_{\infty }}\frac{\partial^{2}T}{\partial y^{2}}, \end{array}$$ (8)

subject to the boundary conditions

$$\begin{array}{c} u=U,\;\;v=0,\;\;T=T_{w},\;\;D_{B}\frac{\partial C}{\partial y}+\frac{D_{T}}{T_{\infty }}\frac{\partial T}{\partial y}=0\;\text{on}\;y=0, \end{array}$$ (9)

$$\begin{array}{c} u\to 0,\;\;T\to T_{\infty },\;\;C=C_{\infty }\;\text{as}\;y\to \infty , \end{array}$$ (10)

where *ν* is the kinematic viscosity, *T* and $\hat{\phi }$ are the local fluid temperature and concentration volume fraction, *T*_∞_ and ${\hat{\phi }}_{\infty }$ are the fluid temperature and ambient concentration volume fraction respectively, *α*_*m*_ is the effective thermal diffusivity, *D*_*B*_ is the Brownian diffusion coefficient, *D*_*T*_ is thermophoresis diffusion coefficient, *τ* = (*ρc*)_*f*_ /(*ρc*)_*P*_ is the ratio between the effective heat capacity of the nanoparticle material and heat capacity of the fluid.

We introduce the following similarity transformations

$$\begin{array}{ccc} \psi \left(x,y,t\right) & = & \sqrt{\frac{b\nu }{1-at}}xf\left(\eta \right),\;\;\;\;\;\;\;\;\;\eta =\sqrt{\frac{b}{\nu (1-at)}}\;y,\\ T\left(x,y,t\right) & = & T_{\infty }+T_{ref}[\frac{bx^{2}}{2\nu }]{\left(1-at\right)}^{-\frac{3}{2}}\theta \left(\eta \right),\\ C\left(x,y,t\right) & = & C_{\infty }+C_{ref}[\frac{bx^{2}}{2\nu }]{\left(1-at\right)}^{-\frac{3}{2}}\phi \left(\eta \right), \end{array}$$

where *θ* and *ϕ* are the dimensionless temperature and nanoparticle volume fraction respectively and *η* is a similarity variable. The physical stream function *ψ*(*x*, *y*, *t*) automatically ensures that mass conservation given in Eq (6). The velocity components are readily obtained as

$$\begin{array}{c} u=\frac{\partial \psi }{\partial y}=\frac{bx}{\left(1-at\right)}f^{\prime }\left(\eta \right),v=-\frac{\partial \psi }{\partial x}=-\sqrt{\frac{\nu b}{\left(1-at\right)}}f\left(\eta \right), \end{array}$$ (11)

where *f*′ is the dimensionless velocity. Eqs (7)–(8) can be presented as

$$\begin{array}{ccc} & & f^{\prime \prime \prime }+ff^{\prime \prime }-f^{\prime 2}-S(f^{\prime }+\frac{1}{2}\eta \;f^{\prime \prime })+\beta_{1}\left(2ff^{\prime }f^{\prime \prime }-f^{2}f^{\prime \prime \prime }\right)+\beta_{2}\left(f^{\prime \prime 2}-ff^{\prime \prime \prime \prime }\right)\\ & & \;+\beta_{2}S(2f^{\prime \prime \prime }+\frac{1}{2}\eta f^{\prime \prime \prime \prime })-\beta_{1}S^{2}\;(2f^{\prime }+\frac{7}{4}\eta f^{\prime \prime }+\frac{1}{4}\eta^{2}f^{\prime \prime \prime })\\ & & \;\;\;-\beta_{1}S(2f^{\prime 2}-3ff^{\prime \prime }+\frac{3}{2}\eta f^{\prime }f^{\prime \prime }-\frac{1}{2}\eta ff^{\prime \prime }-\eta ff^{\prime \prime \prime })=0, \end{array}$$ (12)

$$\begin{array}{c} \theta^{\prime \prime }+Pr[f\theta^{\prime }-2f^{\prime }\theta -\frac{S}{2}\left(3\theta +\eta \theta^{\prime }\right)]+N_{b}\phi^{\prime }\theta^{\prime }+N_{t}\theta^{\prime 2}=0, \end{array}$$ (13)

$$\begin{array}{c} \phi^{\prime \prime }+Le\;[f\phi^{\prime }-2f^{\prime }\phi -\frac{S}{2}\;(3\phi +\eta \phi^{\prime })]+\frac{N_{t}}{N_{b}}\;\theta^{\prime \prime }=0, \end{array}$$ (14)

with the boundary conditions

$$\begin{array}{c} f^{\prime }=1,\;\;f=0,\;\;\theta =1,\;\;N_{b}\phi^{\prime }+N_{t}\theta^{\prime }=0\;\text{at}\;\eta =0, \end{array}$$ (15)

$$\begin{array}{c} f^{\prime }\to 0,\;\;\theta^{\prime }\to 0,\;\;\phi \to 0\;\text{as}\;\eta \to \infty , \end{array}$$ (16)

where *S* is the dimensionless measure of the unsteadiness, *β*_1_ and *β*_2_ are the Deborah numbers in terms of relaxation and retardation times, respectively, the Prandtl number *Pr*, the Brownian motion parameter *N*_*b*_, the thermophoresis parameter *N*_*t*_, the Lewis number *Le*. These parameters are defined as

$$\begin{array}{ccc} S & = & \frac{a}{b},\;\beta_{1}=\delta_{1}b,\;\beta_{2}=\delta_{2}b,\;Pr=\frac{\nu }{\alpha_{m}},\;Le=\frac{\nu }{D_{B}},\\ N_{t} & = & \frac{\tau D_{T}\left(T_{(w}-T_{\infty }\right)}{T_{\infty }\alpha_{m}},\;\;Nb=\frac{\tau D_{B}C_{ref}[\frac{bx^{2}}{2\nu }]}{\alpha_{m}}{\left(1-at\right)}^{-\frac{3}{2}}. \end{array}$$

The non-dimensional form of the Nusselt number and Sherwood number that describe the heat and nanoparticle volume fraction transfer at the surface are

$$\begin{array}{c} Nu_{x}/Re_{x}^{\frac{1}{2}}=-\theta^{\prime }\left(0\right)\;\text{and}\;Shw_{x}/Re_{x}^{\frac{1}{2}}=-\phi^{\prime }\left(0\right), \end{array}$$

where *Re*_*x*_ = *Ux*/*ν* is the local Reynolds number.

## 2 Method of Solution

To solve Eqs (12)–(14) along with the boundary conditions Eqs (15)–(16), the spectral relaxation method (SRM) was used, see Motsa et al. [37–39]. This method is preferred since it has been shown to be accurate and generally easier to use compared to other common numerical methods such as finite differences.

We start by reducing the order of Eq (12) from fourth to third order. To this end, we set *f*′ = *g*, so that Eq (12) becomes

$$\begin{array}{ccc} & & f^{\prime }=g,\\ & & g^{\prime \prime }+fg^{\prime }-g^{2}-S[g+\frac{1}{2}\eta g^{\prime }]+\beta_{1}\left(2fgg^{\prime }-f^{2}g^{\prime \prime }\right)\\ & & \;+\beta_{2}\left(g^{\prime 2}-fg^{\prime \prime \prime }\right)+\beta_{2}S(2g^{\prime \prime }+\frac{1}{2}\eta g^{\prime \prime \prime })-\beta_{1}S^{2}(2g+\frac{7}{4}\eta g^{\prime }+\frac{1}{4}\eta^{2}g^{\prime \prime })\\ & & \;-\beta_{1}S(2g^{2}-3fg^{\prime }+\frac{3}{2}\eta gg^{\prime }-\frac{1}{2}\eta fg^{\prime }-\eta fg^{\prime \prime })=0. \end{array}$$ (17)

The spectral relaxation algorithm decouples the system of governing Eqs (12)–(14). From the decoupled equations an iteration scheme is developed by evaluating linear terms at the current iteration level *r* + 1 and the nonlinear terms at the previous iteration level *r*. Applying the SRM to Eqs (13)–(14) and (17)–(17) gives the following linear ordinary differential equations;

$$\begin{array}{c} a_{1}g_{r+1}^{\prime \prime \prime }+a_{2}g_{r+1}^{\prime \prime }+a_{3}g_{r+1}^{\prime }+a_{4}g_{r+1}+a_{5}=0, \end{array}$$ (18)

$$\begin{array}{c} f_{r+1}^{\prime }=g_{r+1},\;\;\;f_{r+1}\left(0\right)=0, \end{array}$$ (19)

$$\begin{array}{c} \theta_{r+1}^{\prime \prime }+b_{1}\theta_{r+1}^{\prime }+b_{2}\theta_{r+1}+b_{3}=0, \end{array}$$ (20)

$$\begin{array}{c} \phi_{r+1}^{\prime \prime }+c_{1}\phi_{r+1}^{\prime }+c_{2}\phi_{r+1}+c_{3}=0, \end{array}$$ (21)

$$\begin{array}{c} g_{r+1}\left(0\right)=1,\;\theta_{r+1}\left(0\right)=1,\;N_{b}\phi_{r+1}^{\prime }\left(0\right)+N_{t}\theta_{r+1}^{\prime }\left(0\right)=0, \end{array}$$ (22)

$$\begin{array}{c} g_{r+1}\left(\infty \right)=0,\;\theta_{r+1}\left(\infty \right)=0,\;\phi_{r+1}\left(\infty \right)=0, \end{array}$$ (23)

where *a*_*i*_, *b*_*i*_ and *c*_*i*_ (*i* = 1, 2, …) are given by

$$\begin{array}{ccc} a_{1} & = & \beta_{2}(\frac{1}{2}S\eta -f_{r+1}),\\ a_{2} & = & 1-\beta_{1}(f_{r+1}^{2}+\frac{1}{4}S^{2}\eta^{2}-S\eta f_{r+1})+2\beta_{2}S,\\ a_{3} & = & f_{r+1}-\frac{1}{2}S\eta +\beta_{1}(2f_{r+1}g_{r}-\frac{7}{4}S^{2}\eta +3Sf_{r+1}-\frac{3}{2}S\eta g_{r}+\frac{1}{2}S\eta f_{r+1})\\ & & +2\beta_{2}g_{r}^{\prime },\\ a_{4} & = & -[2g_{r}+S-\beta_{1}(2f_{r+1}g_{r}^{\prime }-s^{2}-4Sg_{r}-\frac{3}{2}S\eta g_{r}^{\prime })],\\ a_{5} & = & -[g_{r}^{2}-\beta_{1}(2f_{r+1}g_{r}g_{r}^{\prime }-2Sg_{r}^{2}-\frac{3}{2}S\eta g_{r}g_{r}^{\prime })-\beta_{2}{g_{r}^{\prime }}^{2}], \end{array}$$

$$\begin{array}{ccc} b_{1} & = & Pr(f_{r+1}-\frac{1}{2}S\eta +N_{b}\phi_{r}^{\prime }),\;b_{2}=-Pr(2g_{r+1}+\frac{3}{2}S),\\ b_{3} & = & -N_{t}\theta_{r}^{\prime 2},\;c_{1}=Le(f_{r+1}-\frac{1}{2}S\eta ),\\ c_{2} & = & -Le(2g_{r+1}+\frac{3}{2}S),\;c_{3}=\frac{N_{t}}{N_{b}}\theta_{r}^{\prime \prime }. \end{array}$$

Starting from given initial approximations *f*_0_, *g*_0_, *θ*_0_ and *ϕ*_0_, Eqs (18)–(21) can be solved iteratively using any suitable numerical method. We opt to use the spectral collocation methods for its accuracy. We find the unknown function at collocation points by requiring that Eqs (18)–(21) be satisfied exactly at these points. A convenient set of collocation points is the Gauss-Lobatto points defined by

$$\begin{array}{c} \omega_{j}=\cos\frac{\pi j}{N},\;\;\;\;j=0,1,\ldots ,N. \end{array}$$ (24)

For convenience, in numerical computations the semi-infinite domain is approximated by the truncated domain [0, *L*]. Then using the linear transformation *η* = *L*(*ω* + 1)/2, we convert [0, *L*] into the interval [−1, 1] in which the spectral method can be used, where *L* = *η*_∞_ is a finite number selected to be large enough to represent the behaviour of the flow properties when *η* is very large. The derivatives are defined as

$$\begin{array}{c} \frac{df}{d\eta }=\sum_{k=0}^{N}D_{jk}f\left(\omega_{k}\right)=Df,\;\;\;\;\;j=0,1,\ldots ,N, \end{array}$$ (25)

where *N* + 1 is the number of collocation points, **D** = 2*D*/*L* and f = [*f*(*ω*_0_), *f*(*ω*_1_), …, *f*(*ω*_*N*_)]^*T*^ is the vector of unknown functions at the collocation points. Applying the Chebyshev spectral collocation method to the system Eqs (18)–(21), we obtain the following matrix equations

$$\begin{array}{c} A_{1,r}g_{r+1}=R_{1,r},\;\;g_{r+1}\left(\omega_{N}\right)=1,\;\;g_{r+1}\left(\omega_{0}\right)=0, \end{array}$$ (26)

$$\begin{array}{c} Df_{r+1}=g_{r+1},\;\;f_{r+1}\left(\omega_{N}\right)=0, \end{array}$$ (27)

$$\begin{array}{c} A_{2,r}\theta_{r+1}=R_{2,r},\;\;\theta_{r+1}\left(\omega_{N}\right)=1,\;\;\theta_{r+1}\left(\omega_{0}\right)=0, \end{array}$$ (28)

$$\begin{array}{c} A_{3,r}\phi_{r+1}=R_{3,r},\;\;N_{b}\phi_{r+1}\left(\omega_{N}\right)+N_{t}\theta_{r+1}\left(\omega_{N}\right),\;\;\phi_{r+1}\left(\omega_{0}\right)=0, \end{array}$$ (29)

where

$$\begin{array}{ccc} A_{1,r} & = & \text{diag}\left[a_{1}\right]D^{3}+\text{diag}\left[a_{2}\right]D^{2}+\text{diag}\left[a_{3}\right]D+\text{diag}\left[a_{4}\right]I,\\ R_{1,r} & = & -a_{5}, \end{array}$$ (30)

$$\begin{array}{ccc} A_{2,r} & = & D^{2}+\text{diag}\left[b_{1}\right]D+\text{diag}\left[b_{2}\right]I,\;\;R_{2,r}=-b_{3}, \end{array}$$ (31)

$$\begin{array}{ccc} A_{3,r} & = & D^{2}+\text{diag}\left[c_{1}\right]D+\text{diag}\left[c_{2}\right]I,\;R_{3,r}=-c_{3}. \end{array}$$ (32)

Here I is an (*N* + 1) × (*N* + 1) diagonal matrix, diag[⋅] denotes a diagonal matrix. We choose suitable initial guesses *f*_0_, *g*_0_, *θ*_0_ and *ϕ*_0_ which satisfy the boundary conditions of governing equations as

$$\begin{array}{c} f_{0}=1-e^{-\eta },\;\;g_{0}=e^{-\eta },\;\;\theta_{0}=e^{-\eta },\;\;\phi_{0}=-\frac{N_{t}}{N_{b}}e^{-\eta }. \end{array}$$ (33)

## 3 Results and Discussion

Eqs (12)–(14) along with the boundary conditions Eqs (15)–(16), were solved numerically using both the spectral relaxation method (SRM) and the quasi-linearization method (QLM), see Bellman and Kalaba [40]. Here the QLM has been used as a benchmarking tool to test the accuracy, and hence the reliability of the SRM results.

The results showing the effects of various parameters on the skin-friction coefficient and the heat transfer rate on the unsteady Oldroyd-B nanofluid are given in Tables 1–3.

**Table 1:** Comparison of results for the −*f*′′(0) with *β*_1_ = 0 and *β*_2_ = 0.

				present results		
*S*	Sharidan [41]	Elbashbeshy [47]	Pal [42]	Ord 4	Ord 5	Ord 6
0.0	—	1.0000	—	1.000000	1.000000	1.000000
0.2	—	—	—	1.068015	1.068012	1.068012
0.4	—	—	—	1.134688	1.134686	1.134686
0.6	—	—	—	1.199113	1.199119	1.199119
0.8	1.261042	1.3345	1.261043	1.261039	1.261043	1.261043
1.2	1.377722	1.4535	1.377724	1.377722	1.377724	1.377724
1.4	—	—	—	1.432835	1.432836	1.432836
2.0	1.587362	1.6828	1.587366	1.587365	1.587366	1.587366

**Table 2:** Comparison of the skin friction coefficient −*f*′′(0) for various values of dimensionless unsteadiness *S*, the Deborah numbers *β*_1_ and *β*_2_, when *N*_*t*_ = 0.5, *N*_*b*_ = 0.5, *Pr* = 7 and *Le* = 10.

			SRM			QLM
*S*	*β*_1_	*β*_2_	Ord 5	Ord 6	Ord 7	Ord 8
0.2	0.3	0.4	0.962067	0.962066	0.962066	0.962066
0.4	0.3	0.4	1.014081	1.014081	1.014081	1.014081
0.6	0.3	0.4	1.064909	1.064909	1.064909	1.064909
0.8	0.3	0.4	1.114378	1.114377	1.114377	1.114377
1.0	0.3	0.4	1.162441	1.162441	1.162441	1.162441
1.2	0.3	0.4	1.209120	1.209120	1.209120	1.209120
1.4	0.3	0.4	1.254465	1.254465	1.254465	1.254465
1.6	0.3	0.4	1.298547	1.298546	1.298546	1.298546
0.2	0.1	0.4	0.979541	0.979541	0.979541	0.979541
0.2	0.2	0.4	1.049256	1.049256	1.049256	1.049256
0.2	0.3	0.4	1.114378	1.114377	1.114377	1.114377
0.2	0.4	0.4	1.175721	1.175720	1.175720	1.175720
0.2	0.5	0.4	1.233884	1.233883	1.233883	1.233883
0.2	0.6	0.4	1.289322	1.289320	1.289320	1.289320
0.2	0.7	0.4	1.342389	1.342387	1.342387	1.342387
0.2	0.8	0.4	1.393369	1.393366	1.393366	1.393366
0.2	0.3	0.1	0.979541	0.979541	0.979541	0.979541
0.2	0.3	0.4	1.175721	1.175720	1.175720	1.175720
0.2	0.3	0.7	1.342389	1.342387	1.342387	1.342387
0.2	0.3	1.0	1.489952	1.489947	1.489947	1.489947
0.2	0.3	1.4	1.665997	1.665990	1.665990	1.665990
0.2	0.3	1.7	1.786444	1.786435	1.786435	1.786435
0.2	0.3	2.0	1.899127	1.899117	1.899117	1.899117
0.2	0.3	2.5	2.073154	2.073142	2.073142	2.073142

**Table 3:** Comparison of heat transfer rate −*θ*′(0) for various values of dimensionless unsteadiness *S*, the Deborah numbers *β*_1_ and *β*_2_, when *N*_*t*_ = 0.5, *N*_*b*_ = 0.5, *Pr* = 7 and *Le* = 10.

			SRM			QLM
*S*	*β*_1_	*β*_2_	Ord 5	Ord 7	Ord 8	
0.2	0.3	0.4	4.039516	4.039531	4.039531	4.039531
0.4	0.3	0.4	4.232584	4.232585	4.232585	4.232585
0.6	0.3	0.4	4.418677	4.418690	4.418690	4.418690
0.8	0.3	0.4	4.598443	4.598455	4.598455	4.598455
1.0	0.3	0.4	4.772377	4.772390	4.772390	4.772390
1.2	0.3	0.4	4.940927	4.940940	4.940940	4.940940
1.4	0.3	0.4	5.104494	5.104507	5.104507	5.104507
1.6	0.3	0.4	5.263439	5.263453	5.263453	5.263453
0.2	0.1	0.4	4.620580	4.620593	4.620593	4.620593
0.2	0.3	0.4	4.598443	4.598455	4.598455	4.598455
0.2	0.5	0.4	4.579026	4.579036	4.579036	4.579036
0.2	0.7	0.4	4.561566	4.561574	4.561574	4.561574
0.2	0.5	0.1	4.620580	4.620593	4.620593	4.620593
0.2	0.5	0.4	4.588452	4.588463	4.588463	4.588463
0.2	0.5	0.7	4.561566	4.561574	4.561574	4.561574
0.2	0.5	1.0	4.538087	4.538091	4.538091	4.538091

Table 1 gives a comparison between the present results and the results obtained by Sharidan [41] and Pal [42] for the skin friction. There is a good agreement between the two sets of results with the SRM having converged at the fifth order up to six decimal places. The SRM results are further validated by comparison with results generated using a quasilinearisation method. The quasilinearisation method has also been used recently, albeit in a slightly different form, by Ibrahim and Shanker [43]

Table 2 shows a further comparison of the spectral relaxation and quasilinearisation results for the skin friction coefficient while Table 3 shows the variation of the heat transfer rate for different values of dimensionless unsteadiness parameter and Deborah numbers. The comparison of the two methods shows an excellent agreement between the numerical results obtained by the spectral relaxation and the quasilinearisation methods. In addition, Table 2 shows that the skin friction coefficient increases with increasing values of the unsteadiness parameter *S* and the relaxation time in terms of the Deborah number *β*_1_ and decreases with increasing retardation time or Deborah number *β*_2_. The heat transfer coefficient −*θ*′(0) is however shown to decrease with *β*_1_ in Table 3. he increase in the skin friction coefficient with the flow unsteadiness has also been observed in earlier studies, such as in Ibrahim and Shanker [43] and Mukhopadhyay et al. [44]. It has been suggested in Mukhopadhyay et al. [44] that a decrease in the skin friction coefficient may be important in coating processes where higher stretching speeds may be achieved for smaller pulling forces. The study by Mukhopadhyay et al. [44] was on unsteady flow in a Casson fluid and further showed that for Casson fluids, the temperature decreased significantly with unsteadiness.

We observe further that heat transfer coefficient increases with increased unsteadiness and *β*_2_. The negative values of the nanoparticle profile are due to the fact that the effect of thermophoresis is such that an elevation above the ambient surface temperature leads to a reduction in the relative value of the nanoparticle fraction at the surface (see Kuznetsov and Nield [34]).

**Fig 2:**
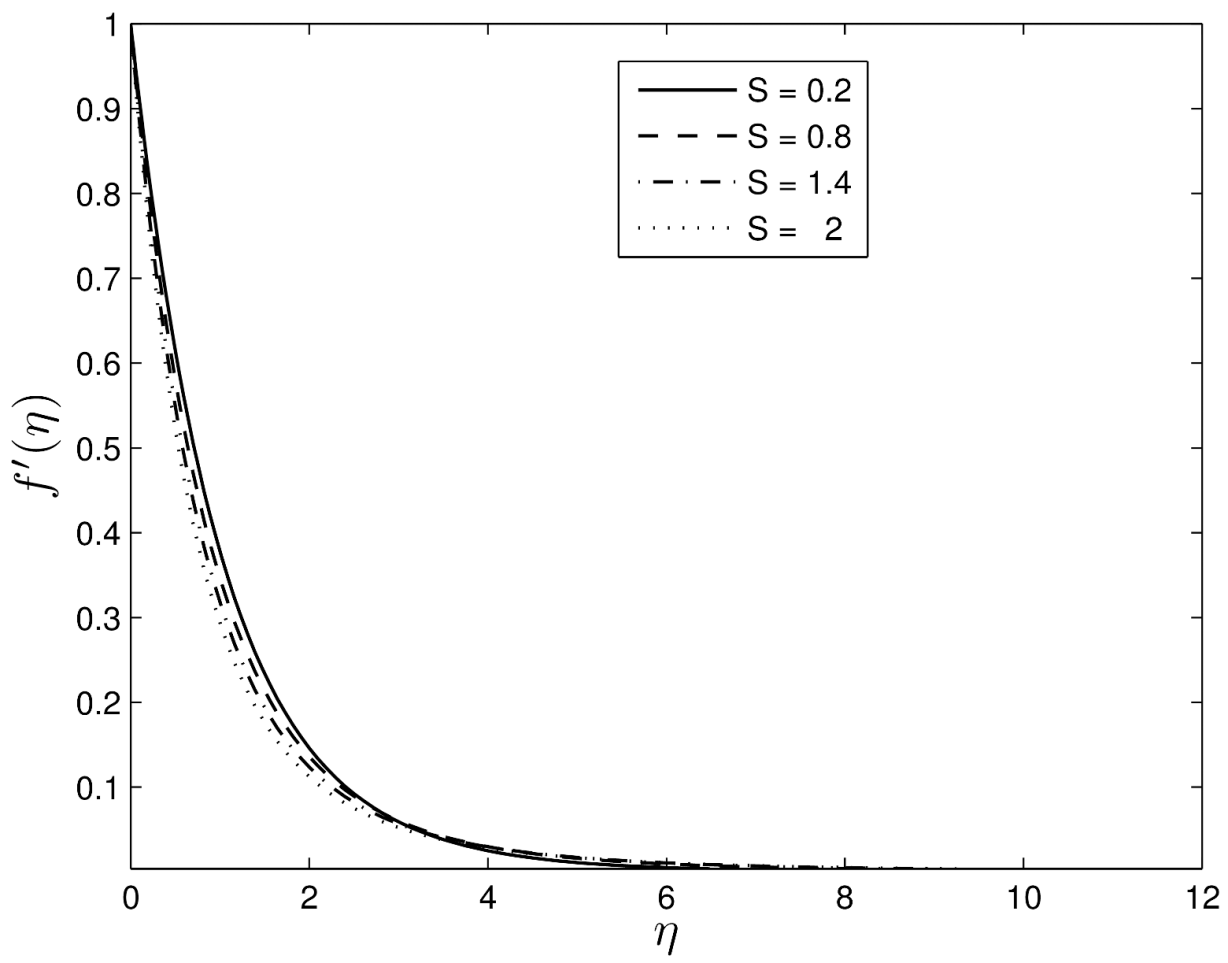
Effect of the unsteadiness parameter *S* on the velocity *f*′(*η*) when *β*_1_ = 0.3, *β*_2_ = 0.4 *Le* = 10, *Pr* = 5, *Nt* = 0.5 and *Nb* = 0.5.

**Fig 3:**
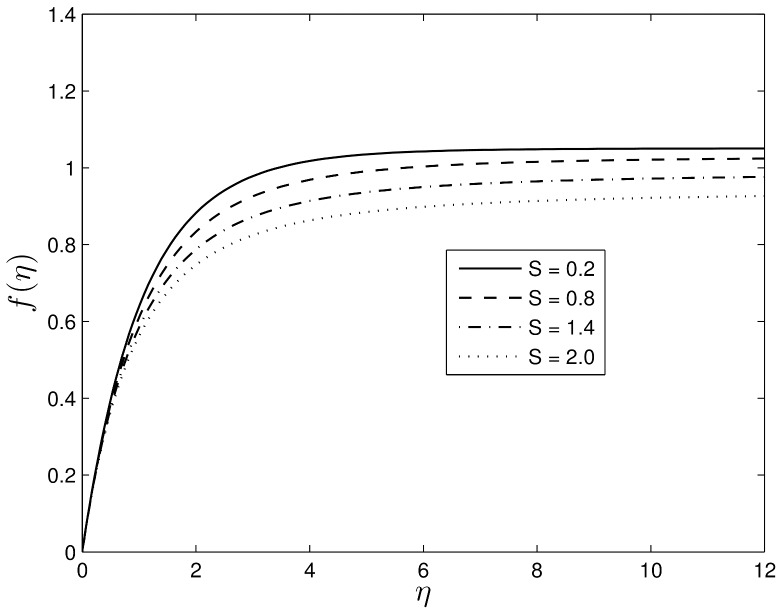
Effect of the unsteadiness parameter *S* on the velocity *f*′(*η*), when *β*_1_ = 0.3, *β*_2_ = 0.4 *Le* = 10, *Pr* = 5, *Nt* = 0.5 and *Nb* = 0.5.

**Fig 4:**
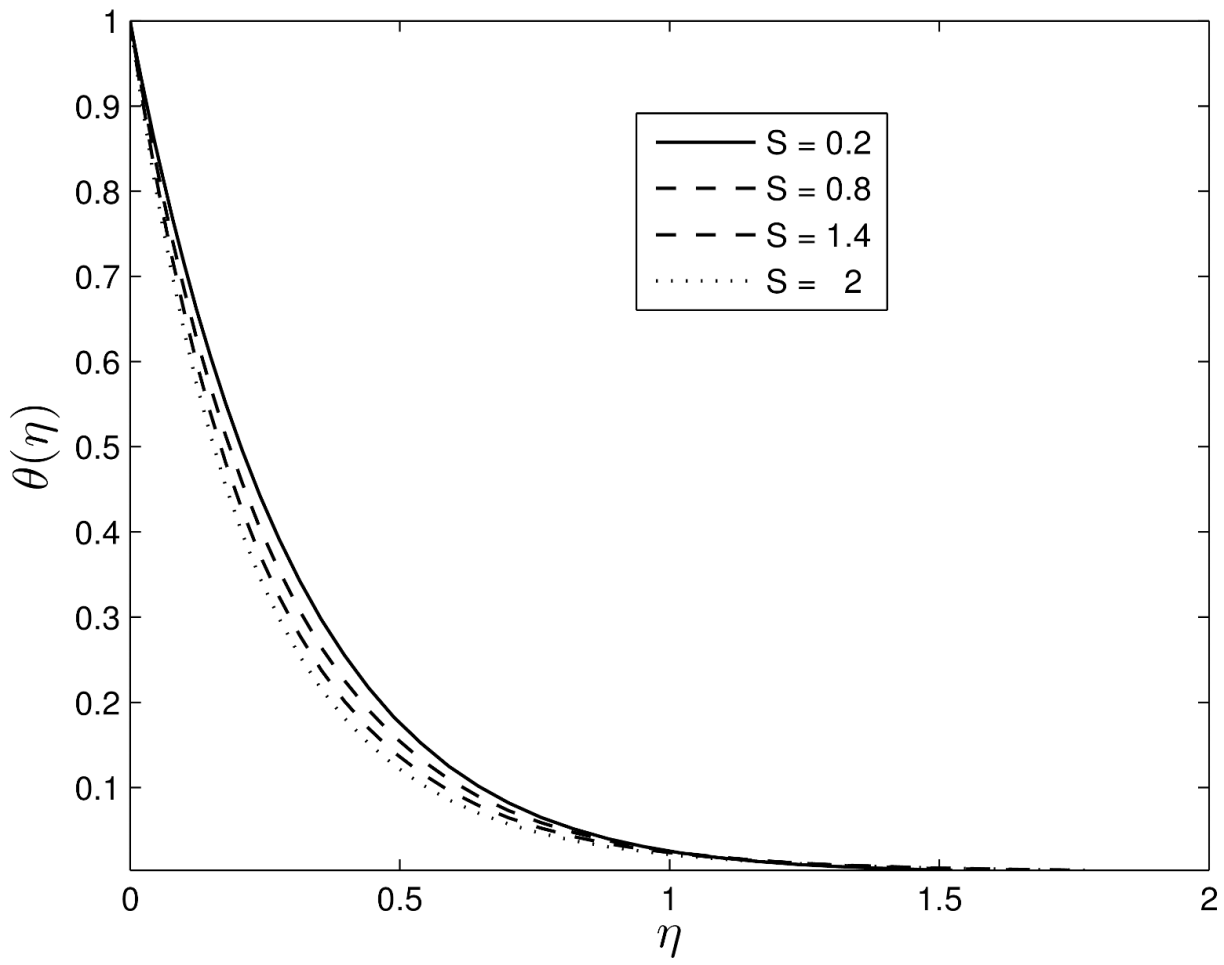
Effect of the unsteadiness parameter *S* on *θ*(*η*) when *β*_1_ = 0.3, *β*_2_ = 0.4 *Le* = 10, *Pr* = 5, *Nt* = 0.5 and *Nb* = 0.5.

**Fig 5:**
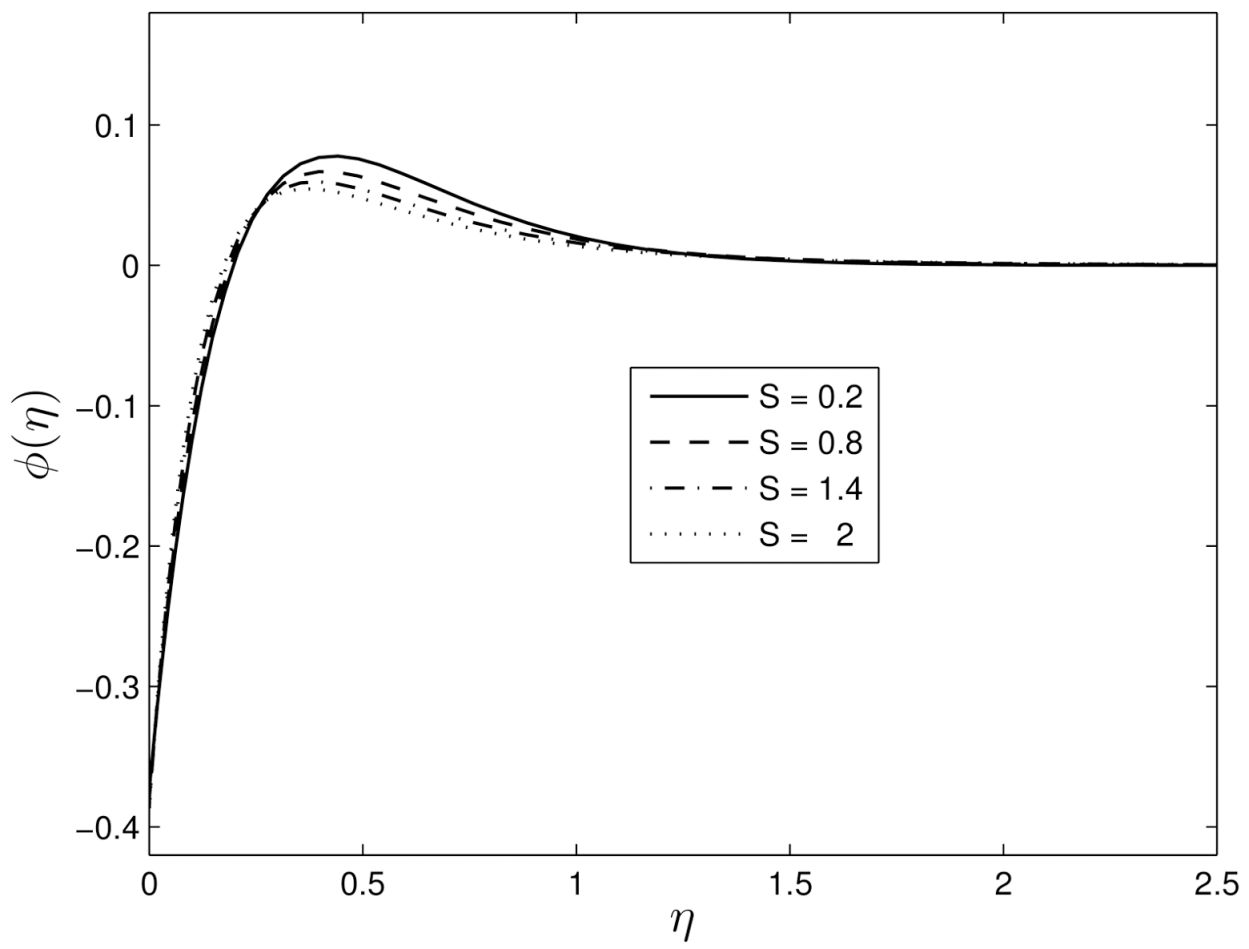
Effect of the unsteadiness parameter *S* on *ϕ*(*η*) for *β*_1_ = 0.3, *β*_2_ = 0.4 *Le* = 10, *Pr* = 5, *Nt* = 0.5 and *Nb* = 0.5.

Figs 2 and 3 show the velocity profiles for different values of the unsteadiness parameter. We observe that the velocity distribution and the momentum boundary layer thicknesses reduce with an increase in the unsteadiness parameter. This finding is in line with the earlier findings of Ibrahim and Shanker [43], and shows that even in the absence of an applied magnetic field, the velocity profiles decrease with the unsteadiness parameter. Figs 4 and 5 show the dimensionless temperature and concentration volume fraction profiles respectively for selected values of *S*. The steepness in both the temperature and concentration profiles decreases reducing the thicknesses of both the thermal and concentration volume fraction boundary layers. These results also follow a similar trend as observed by Ibrahim and Shanker [43] and Mukhopadhyay et al. [44]. Further, it may be pointed out that the concentration volume fraction increases from negative to positive values until boundary layer separation occurs. The magnitude of the volume fraction concentration increases up to a critical point and then decreases to zero.

**Fig 6:**
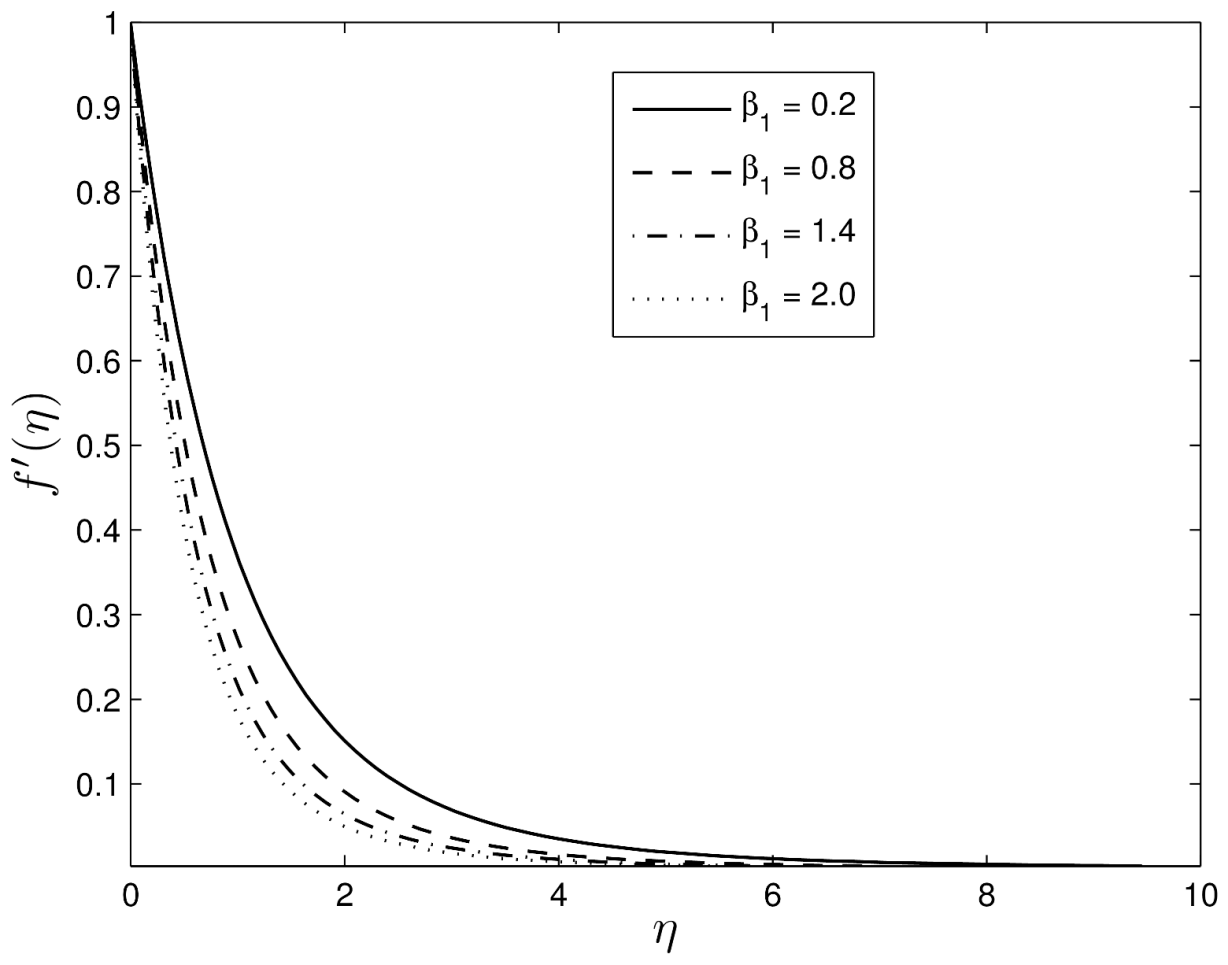
Effect of *β*_1_ on velocity component *f*′(*η*) for *S* = 0.8, *Le* = 10, *Pr* = 5, *Nt* = 0.5 and *Nb* = 0.5.

**Fig 7:**
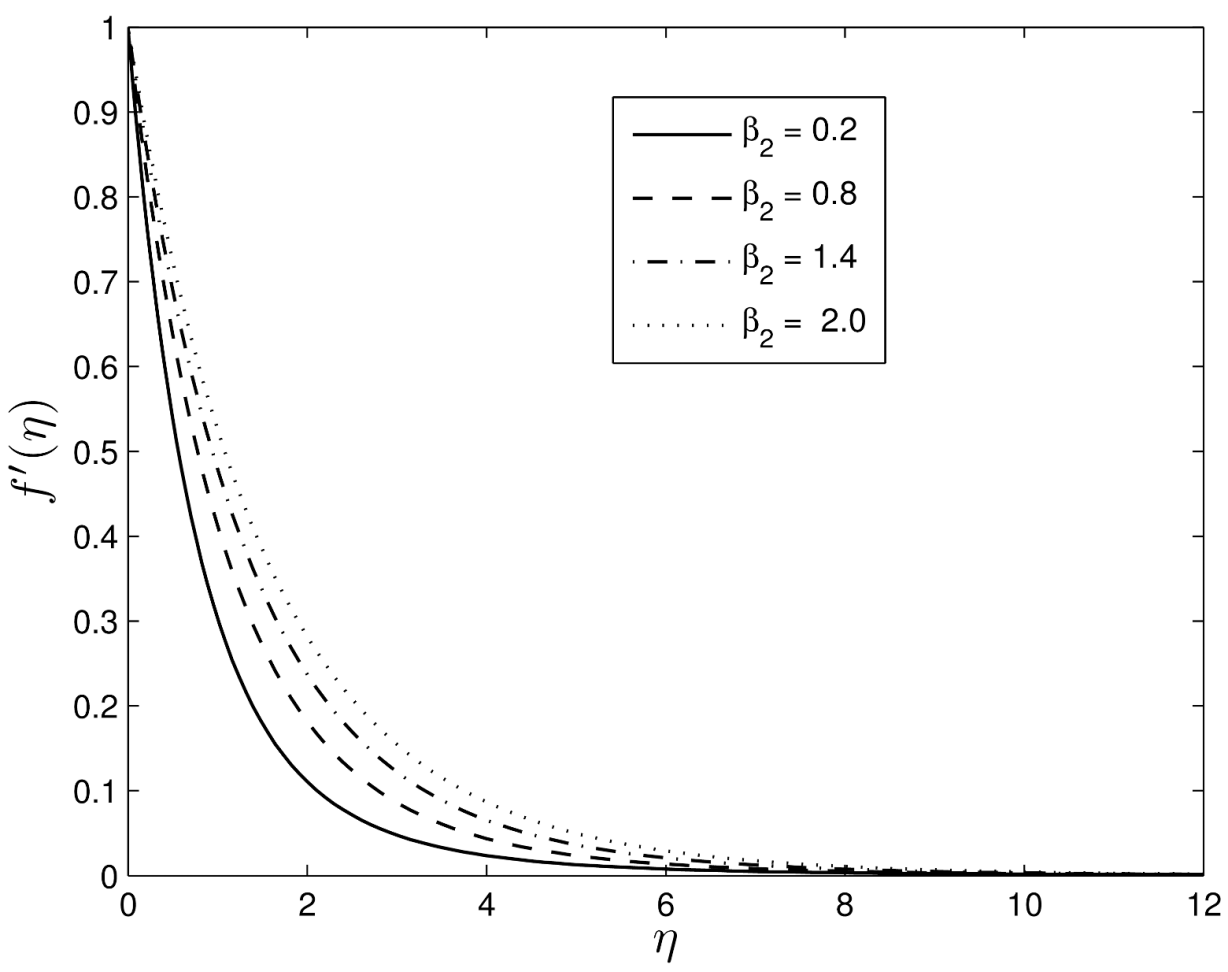
Effect of *β*_2_ on velocity component *f*′(*η*) for *S* = 0.8, *Le* = 10, *Pr* = 5, *Nt* = 0.5 and *Nb* = 0.5.

Figs 6 and 7 show the influence of the Deborah numbers *β*_1_ and *β*_2_ on the velocity profiles. Higher Deborah numbers are indicative that the Oldroyd-B nanofluid is stretched. The nanofluid velocity *f*′(*η*) and the momentum boundary layer thickness decrease with increasing *β*_1_ which is not an unexpected result since it is well known that the viscoelastic fluid resists the motion of the fluid.

**Fig 8:**
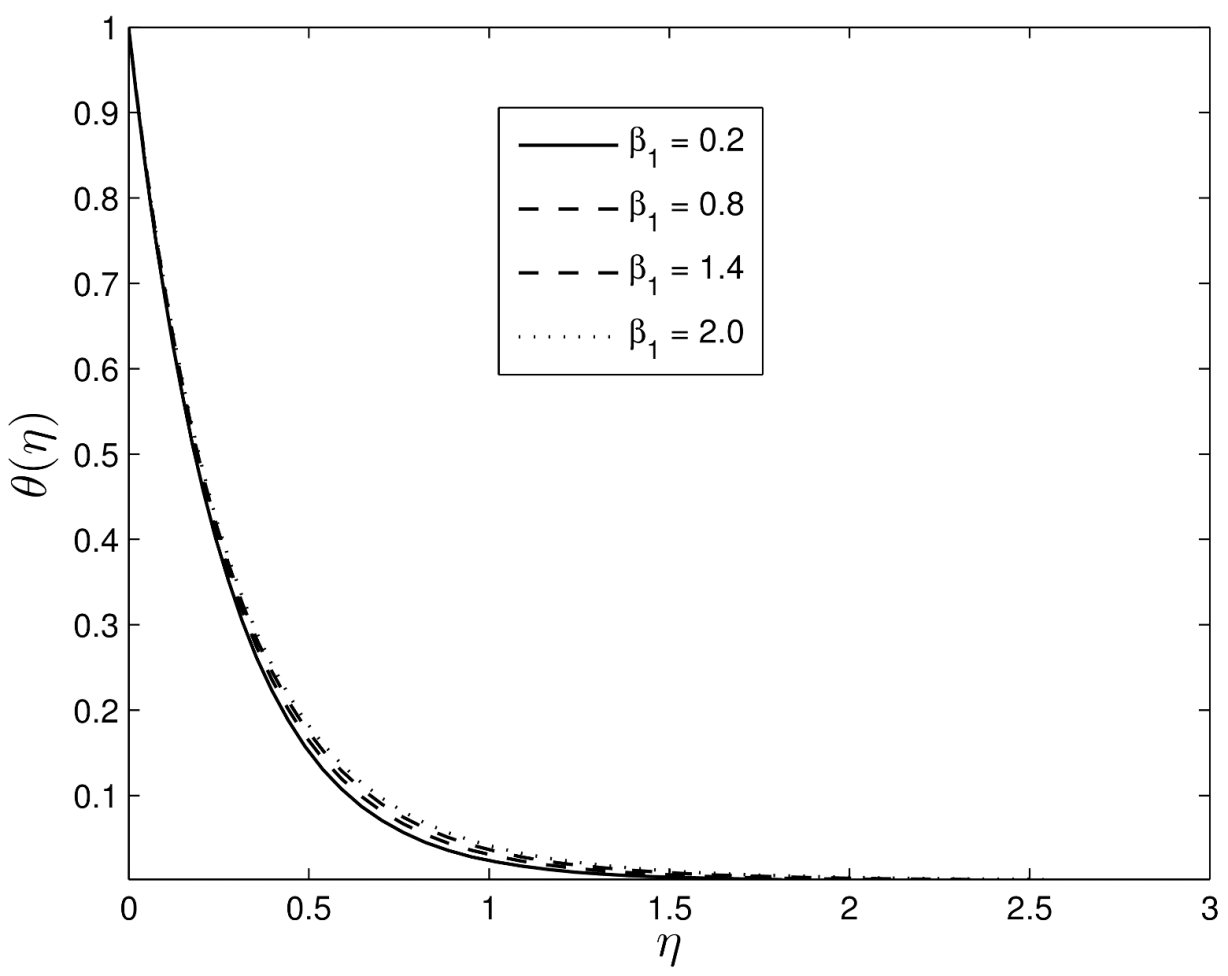
Effect of the unsteadiness parameter *β*_1_ on *θ*(*η*) for *S* = 0.8, *β*_2_ = 0.4 *Le* = 10, *Pr* = 5, *Nt* = 0.5 and *Nb* = 0.5.

**Fig 9:**
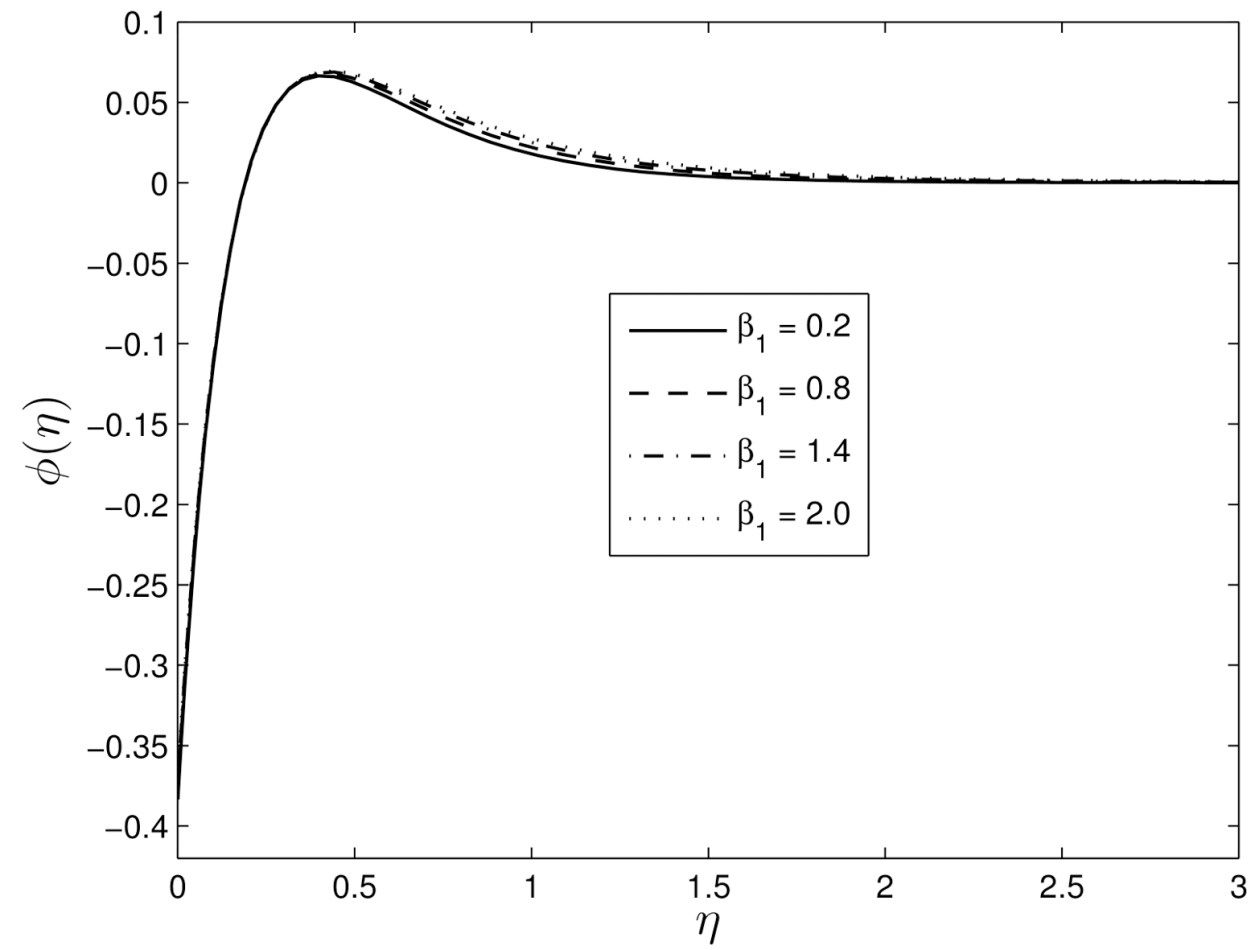
Effect of the unsteadiness parameter *β*_1_ on *ϕ*(*η*) for *S* = 0.8, *β*_2_ = 0.4 *Le* = 10, *Pr* = 5, *Nt* = 0.5 and *Nb* = 0.5.

Figs 8 and 9 show the effects of *β*_1_ on the temperature and concentration profiles, respectively. As *β*_1_ increases, both the nanofluid temperature and the concentration volume fraction increase enhancing both the thermal and the concentration boundary layer thicknesses.

**Fig 10:**
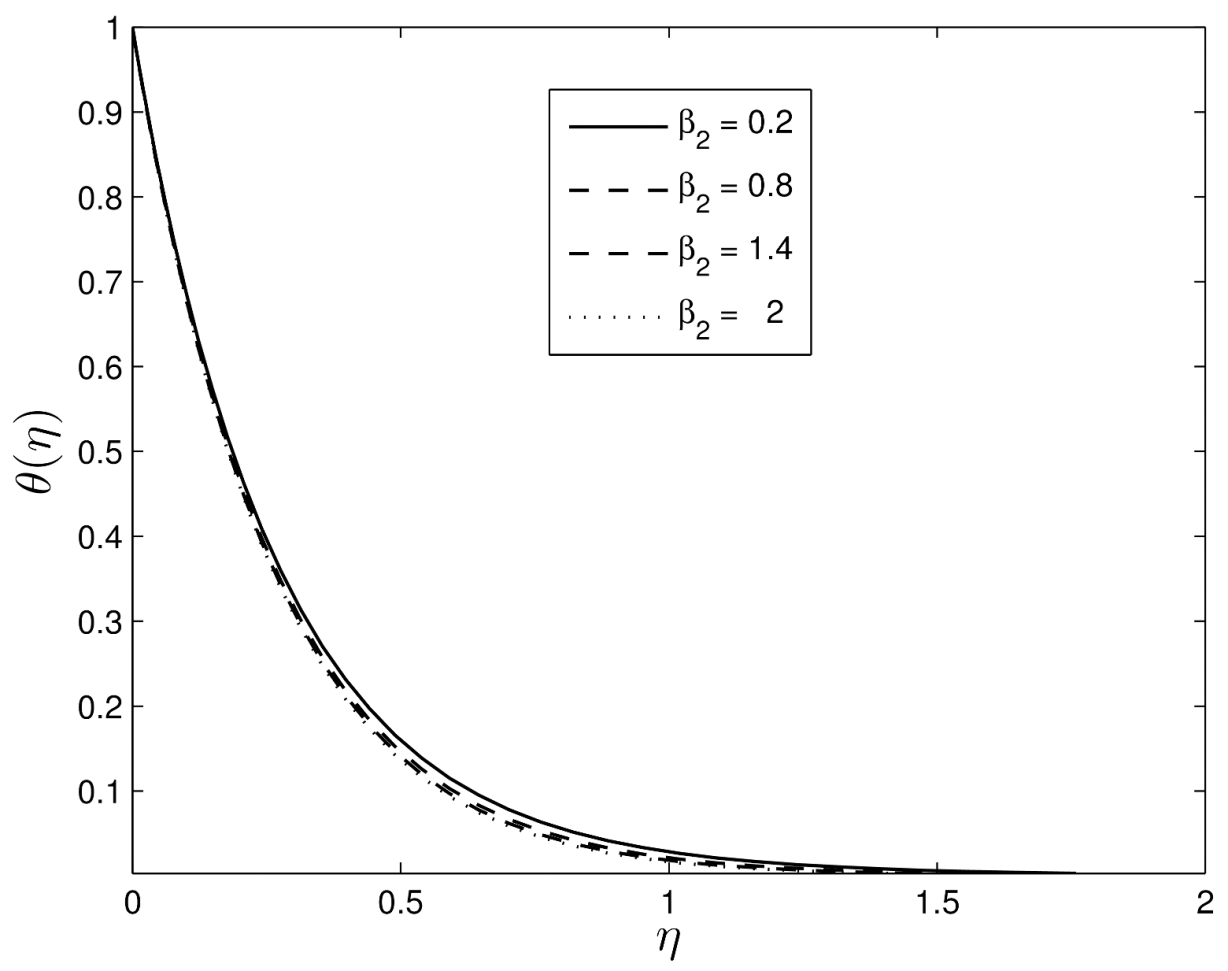
Effect of the unsteadiness parameter *β*_2_ on *θ*(*η*) for *β*_1_ = 0.3, *S* = 0.8 *Le* = 10, *Pr* = 5, *Nt* = 0.5 and *Nb* = 0.5.

**Fig 11:**
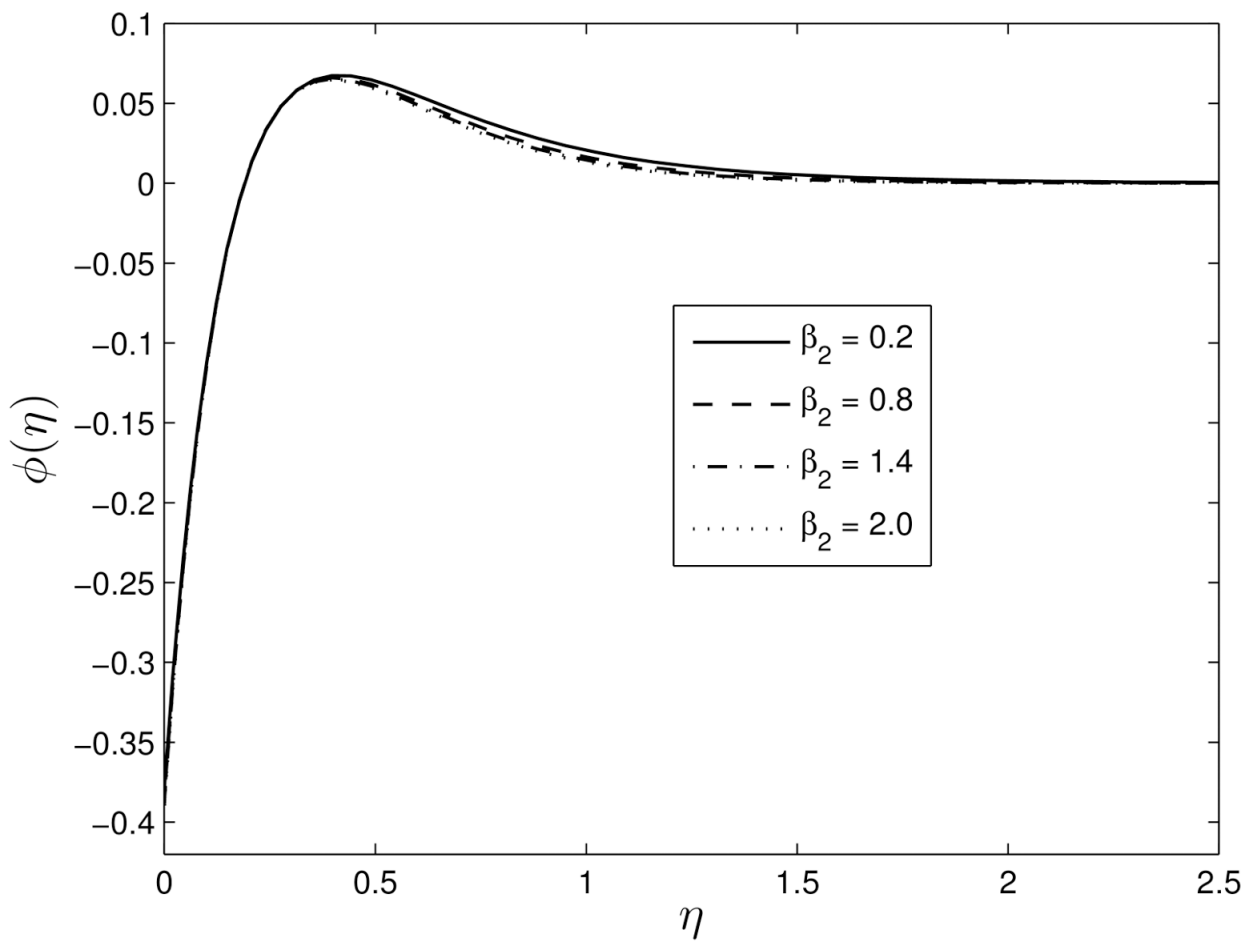
Effect of the unsteadiness parameter *β*_2_ on *ϕ*(*η*) for *β*_1_ = 0.3, *S* = 0.8 *Le* = 10, *Pr* = 5, *Nt* = 0.5 and *Nb* = 0.5.

Figs 10 and 11 show the variation of the temperature and concentration volume fraction profiles for difference values of the retardation Deborah number *β*_2_. It can be seen that when *β*_2_ increases, both the temperature and concentration volume fraction distributions decrease thus diminishing the thicknesses of both the thermal and volume fraction boundary layers.

**Fig 12:**
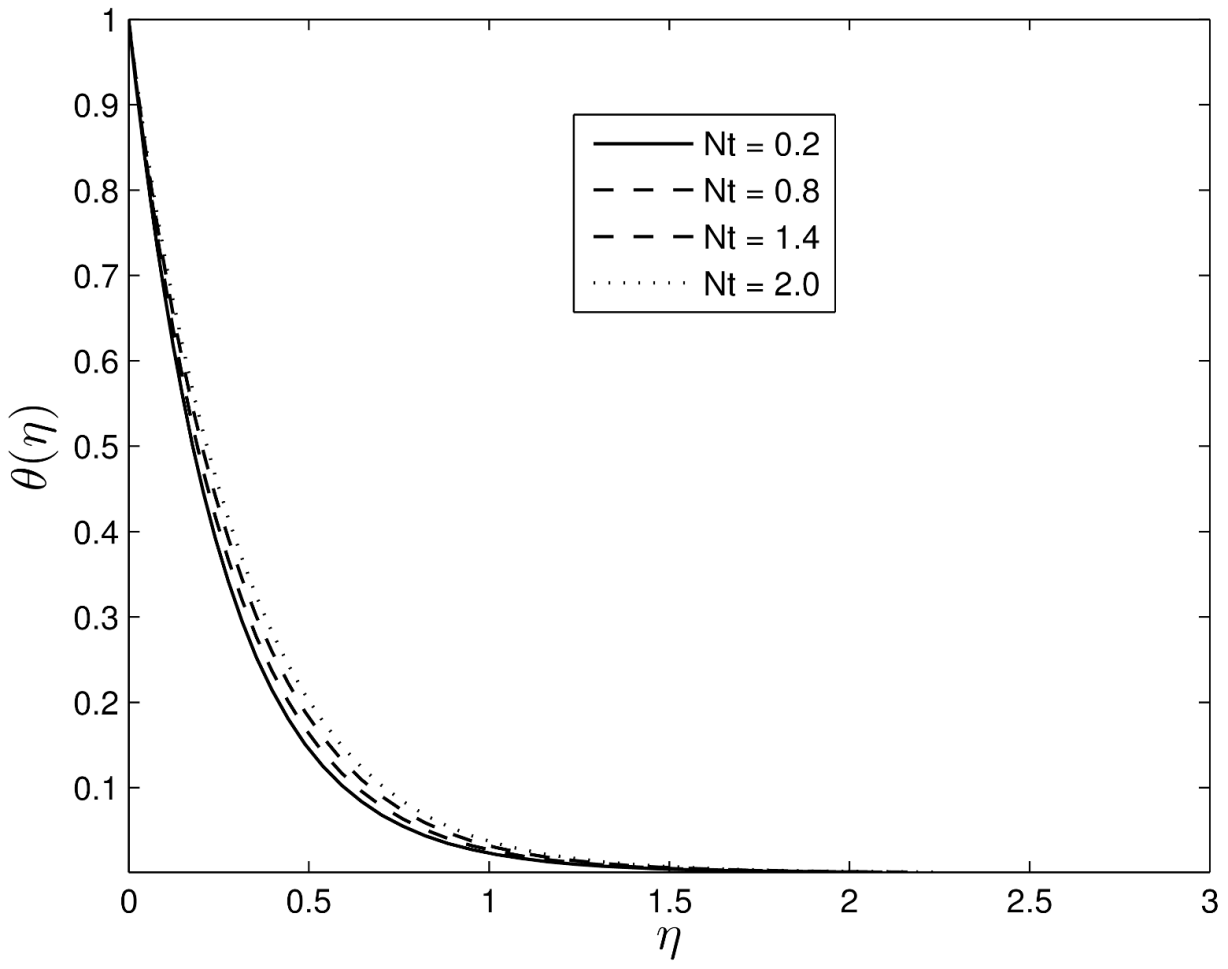
Effect of the thermophoresis parameter *Nt* on *θ*(*η*) for *S* = 0.2, *β*_1_ = 0.3, *β*_2_ = 0.4 *Le* = 10, *Pr* = 5 and *Nb* = 0.5.

**Fig 13:**
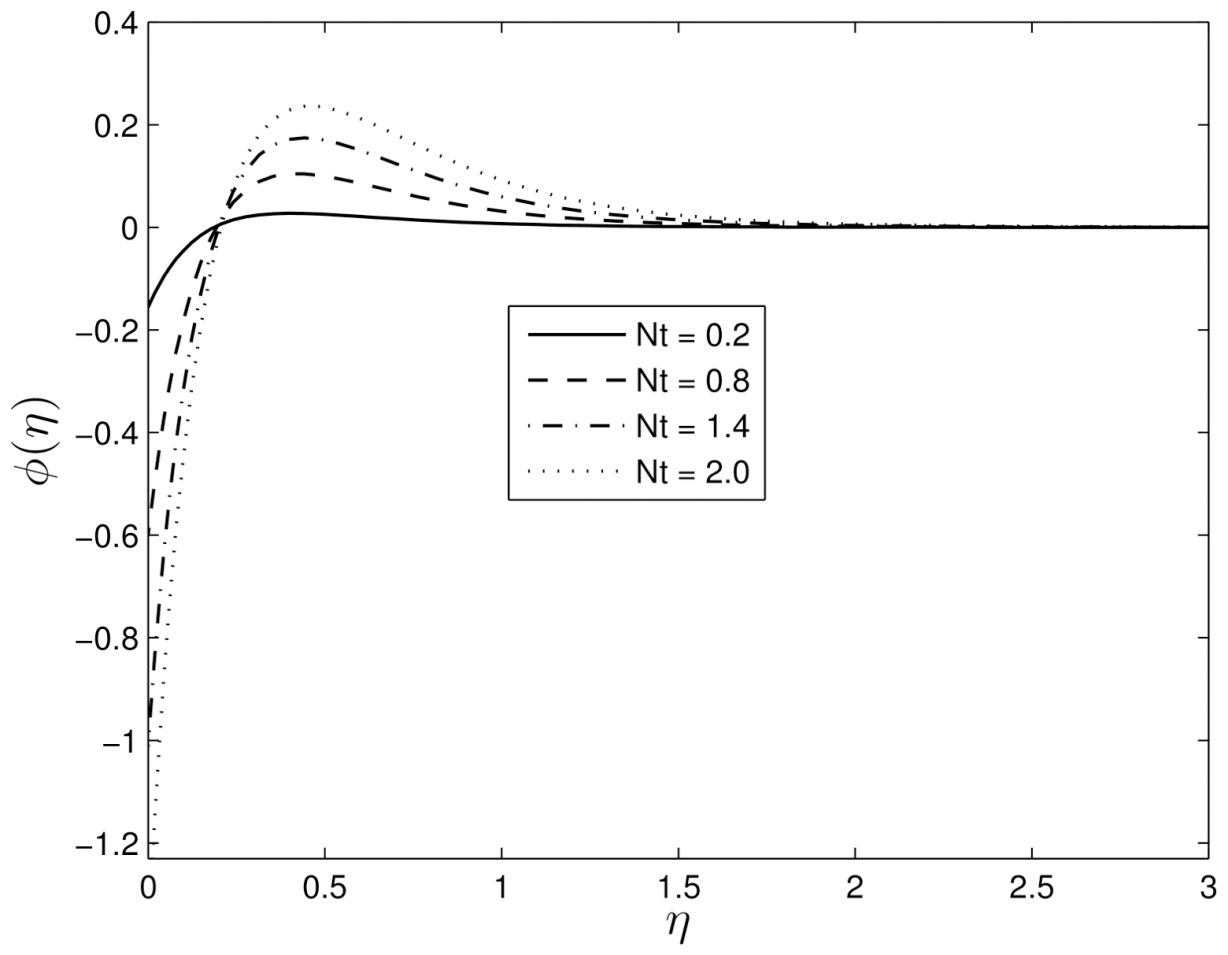
Effect of thermophoresis *Nt* on *ϕ*(*η*) for *S* = 0.2, *β*_1_ = 0.3, *β*_2_ = 0.4 *Le* = 10, *Pr* = 5 and *Nb* = 0.5.

Figs 12 and 13 show the effect of the thermophoresis parameter *Nt* on *θ*(*η*) and *ϕ*(*η*) for fixed *S*, *β*_1_, *β*_2_, *Le* and *Nb*. The temperature gradients in the boundary layer induces a thermophoretic force on the nanoparticles and that leads to a fast flow away from the stretching surface. Hence more fluid is heated away from the surface, and consequently, as *N*_*t*_ increases, the temperature within the boundary layer increases. The fast flow from the stretching sheet carries with it nanoparticles leading to an increase in the mass volume fraction boundary layer thickness. It can also be observed that with an increase in the thermophoretic force, the nanoparticle fraction concentration profiles increase in the boundary layer before reducing to zero far from the surface. Here boundary layer separation occurs early at the stretching surface.

**Fig 14:**
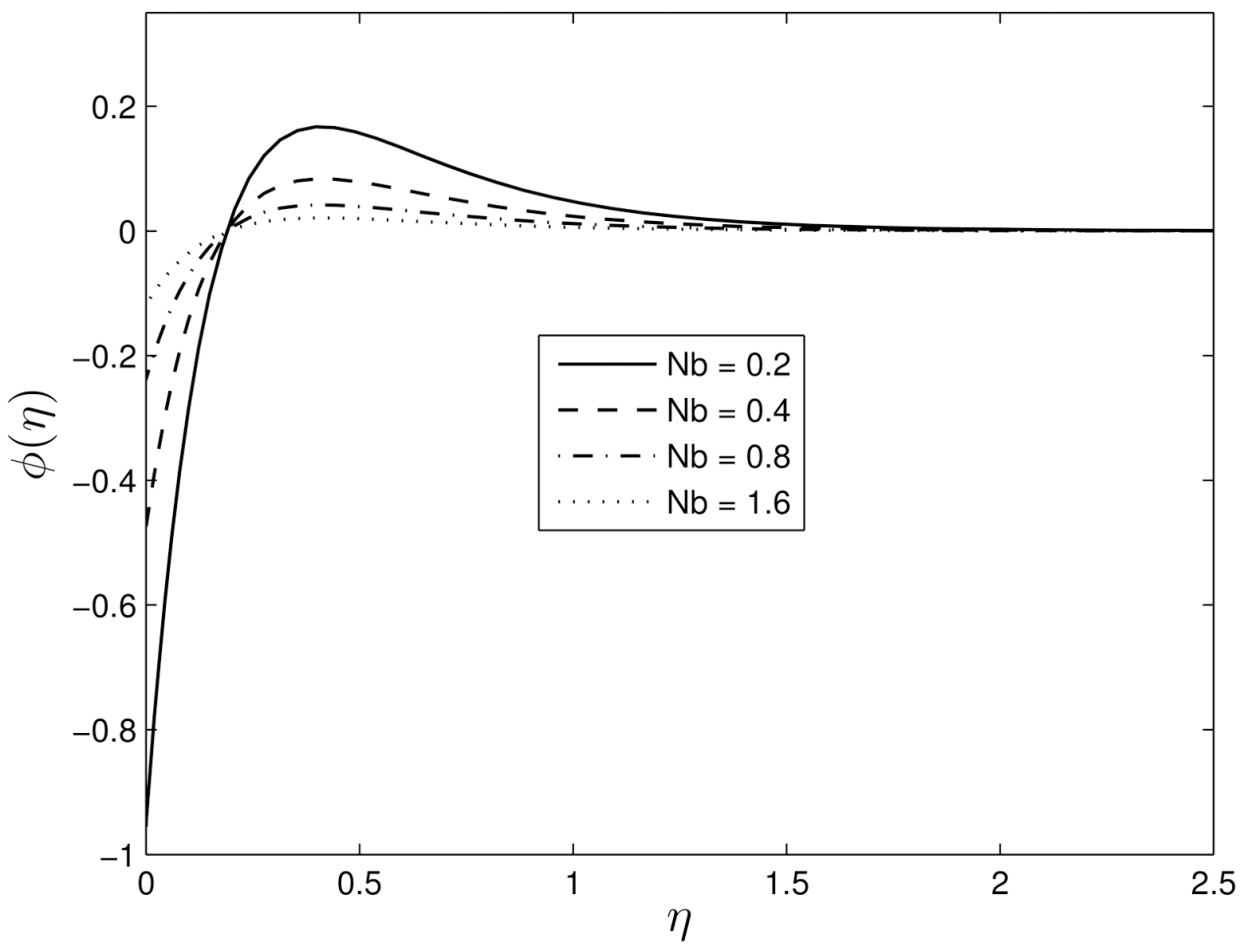
Effect of the Brownian motion *Nb* on *ϕ*(*η*) for *β*_1_ = 0.3, *β*_2_ = 0.4 *Pr* = 7, *Nt* = 0.5.

**Fig 15:**
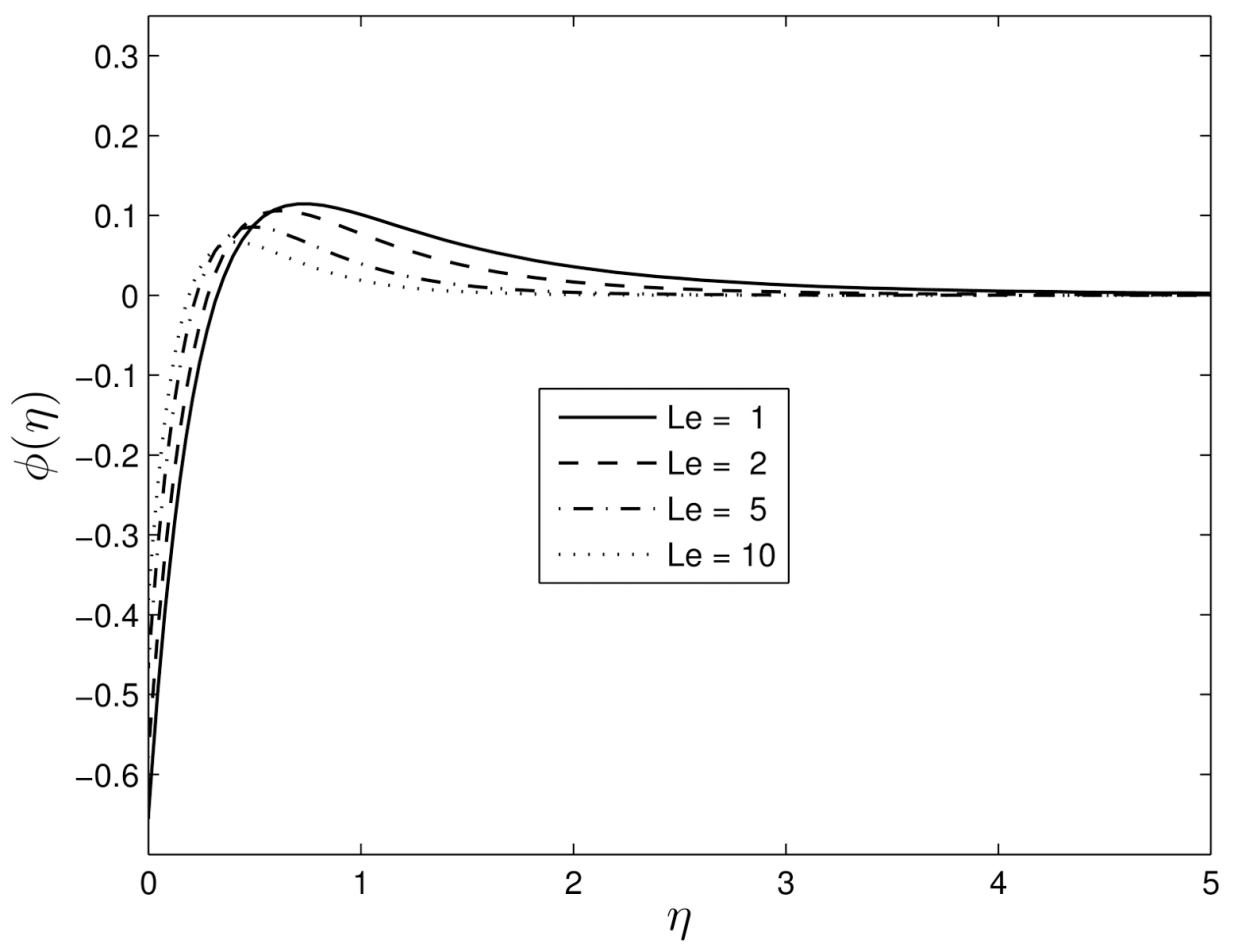
Effect of the Lewis number *Le* on *ϕ*(*η*) for *β*_1_ = 0.3, *β*_2_ = 0.4 *Pr* = 7, *Nt* = 0.5.

Figs 14 and 15 show the influences of the random particle motion (represented by the parameter *N*_*b*_) and the Lewis number *Le* on the nanoparticle volume fraction profiles. The nanoparticle Brownian motion at the molecular level plays a significant role in determining the thermal behaviour of the nanoparticle-fluid suspensions, Jang and Choi [45]. It is obvious that the nanoparticle volume fraction increases close to the stretching surface with increased Brownian motion and Lewis numbers before the boundary layer separation point. However, after the separation point the concentration volume fraction profiles decrease with an increase in both *N*_*b*_ and *Le*.

**Fig 16:**
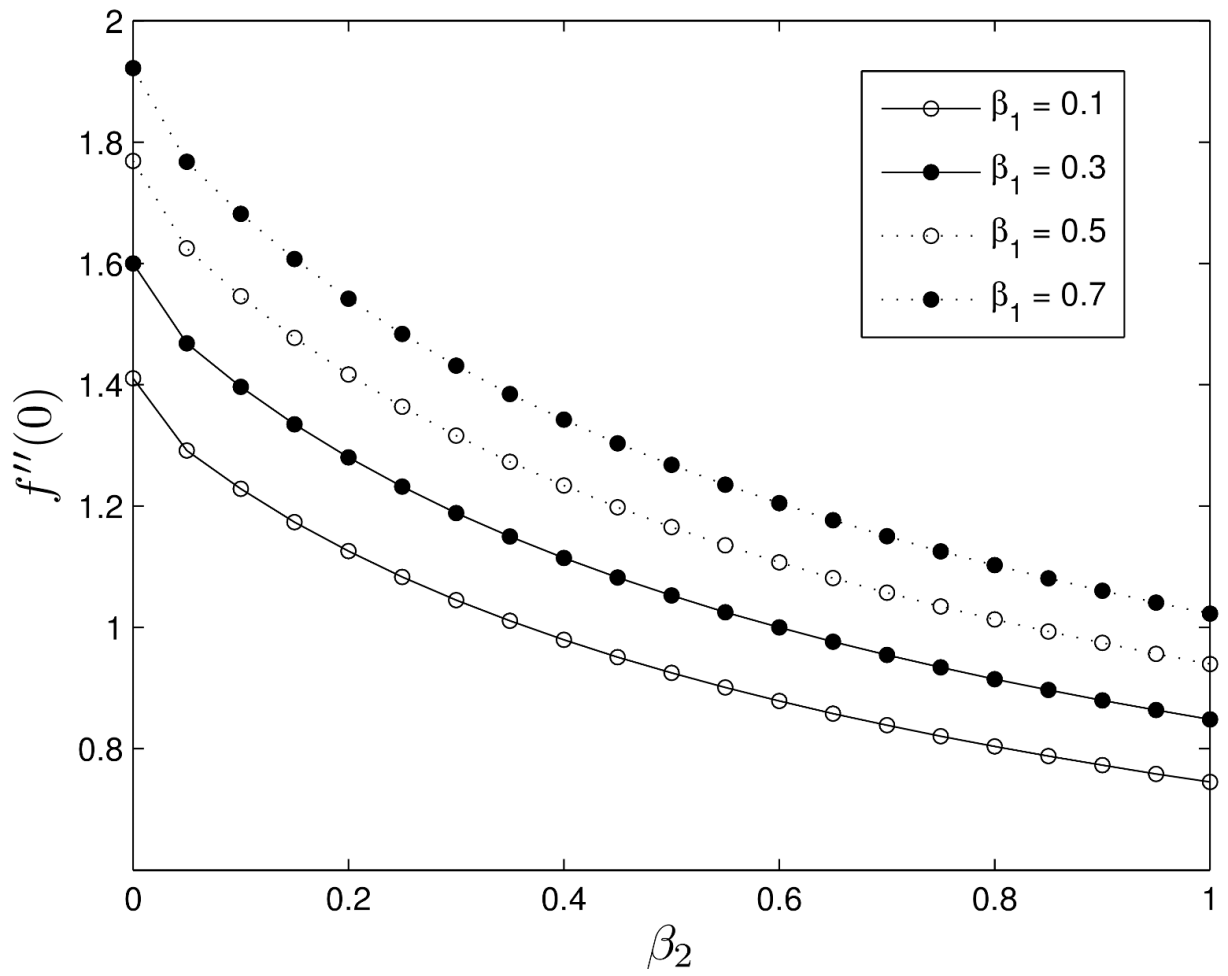
Effect of the Deborah numbers *β*_1_ and *β*_2_ on *f*′′(0) for *S* = 0.8, *Le* = 10, *Pr* = 5, *Nt* = 0.5 and *Nb* = 0.5.

**Fig 17:**
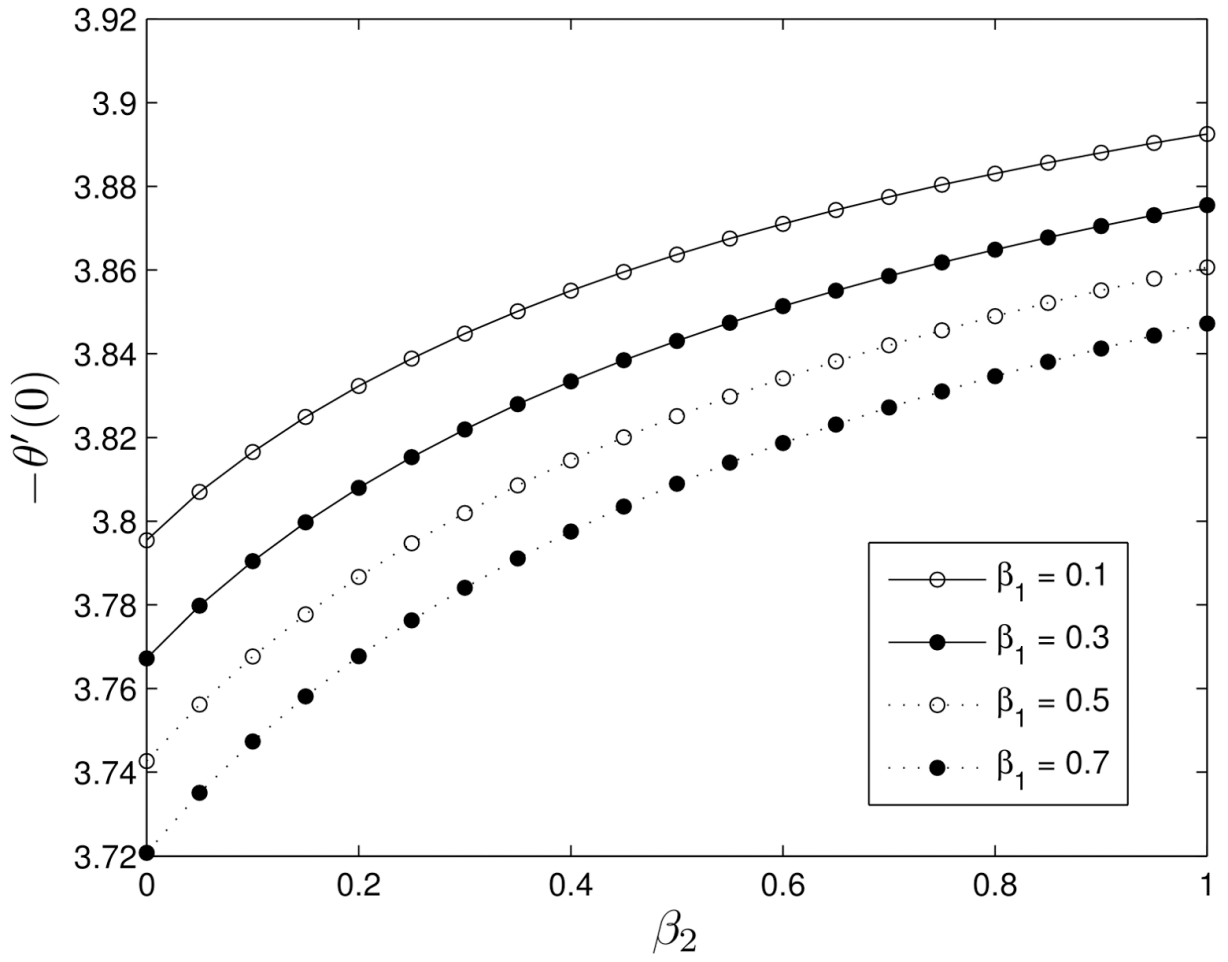
Effect of the Deborah numbers *β*_1_ and *β*_2_ on −*θ*′(0) for *S* = 0.8, *Le* = 10, *Pr* = 5, *Nt* = 0.5 and *Nb* = 0.5.

Figs 16 and 17 show the effects of Deborah numbers *β*_1_ and *β*_2_ on skin friction and heat transfer coefficients. The skin friction coefficient increases with increasing *β*_1_ leading to higher surface shear stresses, while increases in the skin-friction coefficient takes place with increasing *β*_2_. We observe that increasing *β*_1_ leads to a decrease in −*θ*′(0) whereas increasing *β*_2_ enhances the rate of heat transfer.

**Fig 18:**
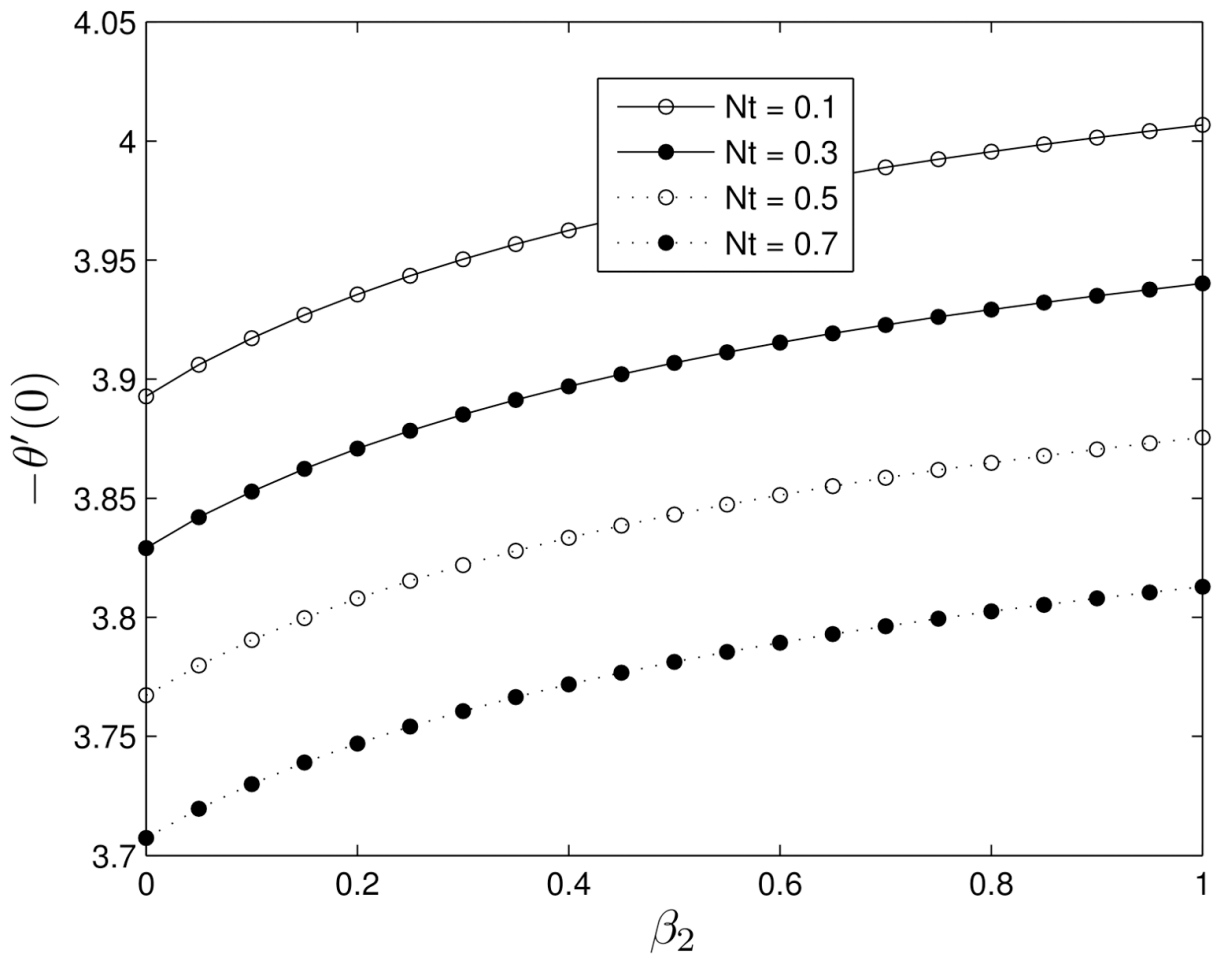
Effect of the thermophoresis number *Nt* on −*θ*′(0) for *β*_1_ = 0.3, *Le* = 10, *Pr* = 5, *Nt* = 0.5 and *Nb* = 0.5.

The variation of heat transfer coefficients with the thermophoresis parameter *Nt* is shown in Fig 18. It is clear that the thermal boundary layer thickness increases when the thermophoresis parameter *N*t increases, and hence reducing the rate of heat transfer.

In order to apply the linear regression formula used by Kuznetsov and Nield [46], we used a set of 125 values of *S*, *Nb* and *Nt* with each *S*, *Nb*, *Nt* restricted to the space [0, 0.5] with a maximum error of less than 1%. Table 4 shows the linear regression coefficients and error bounds for the reduced Nusselt number. Here *C*_*s*_, *C*_*b*_, *C*_*t*_, are the coefficients in the linear regression estimate

$$\begin{array}{c} Nu_{est}/Ra_{x}^{1/2}=Nu_{PKB}+C_{s}S+C_{b}Nb+C_{t}Nt, \end{array}$$

and *ɛ* is the maximum relative error defined by *ɛ* = ∣(*Nu*_*est*_−*Nu*)/*Nu*∣. In this study, the minimum error occurs for small values of *S*, *Nb* and *Nt*.

**Table 4:** Linear regression coefficients and error bounds for the reduced Nusselt number.

*C*_*s*_	*C*_*b*_	*C*_*t*_	*Pr*	*ɛ*
-0.347	-0.111	-0.225	1	0.001
-0.360	-0.128	-0.239	2	0.001
-0.390	-0.141	-0.251	5	0.001
-0.400	-0.167	-0.270	10	0.001
-0.412	-0.197	-0.281	100	0.001

## 4 Conclusions

In this paper we have studied the unsteady Oldroyd nanofluid flow over stretching surface. The classical boundary condition in which both the nanoparticle volume fraction and the temperature are actively controlled has been substituted by the more realistic condition where the nanoparticle volume fraction is not controlled an the boundary. The effects of the governing parameters such as the unsteadiness parameter, the Deborah numbers in terms of relaxation and retardation times, the Prandtl number, the Brownian motion parameter, the thermophoresis parameter, the Lewis number on skin friction, heat transfer coefficients and fluid flow characteristics have been studied. Here *β*_1_ represents the viscoelastic properties of the fluid and resists the motion of the fluid. The effects of the Brownian motion on the rate of heat transfer are negligible. The comparison between results obtained using the SRM and the QLM for skin friction and heat transfer coefficients showed a good agreement, with the SRM having converged at the sixth order up to six decimal places.
